# Novel oxygen saturation imaging endoscopy to assess anastomotic integrity in a porcine ischemia model

**DOI:** 10.1186/s12893-020-00913-6

**Published:** 2020-10-22

**Authors:** Hiro Hasegawa, Nobuyoshi Takeshita, Masaaki Ito

**Affiliations:** 1 Department of Colorectal Surgery, National Cancer Center Hospital East, 6-5-1 Kashiwanoha, Kashiwa, Chiba 277-8577 Japan; 2 Surgical Device Innovation Office, NEXT Medical Device Innovation Center, National Cancer Center Hospital East, Kashiwa, Japan

**Keywords:** Anastomotic integrity, Endoscopy, Perfusion assessment, Tissue oxygen saturation

## Abstract

**Background:**

Establishing anastomotic integrity is crucial for avoiding anastomotic complications in colorectal surgery. This study aimed to evaluate the safety and feasibility of assessing anastomotic integrity using novel oxygen saturation imaging endoscopy in a porcine ischemia model.

**Methods:**

In three pigs, a new endoscope system was used to check the mechanical completeness of the anastomosis and capture the tissue oxygen saturation (StO_2_) images. This technology can derive the StO_2_ images from the differences in the absorption coefficient in the visible light region between oxy- and deoxy-hemoglobin. Bowel perfusion at the proximal rectum was assessed before and after the anastomosis, and 1 min and 30 min after the ligation of the cranial rectal artery (CRA).

**Results:**

The completeness of the anastomoses was confirmed by the absence of air leakage. Intraluminal oxygen saturation imaging was successfully performed in all animals. There was no significant difference in the StO_2_ level before and after the anastomosis (52.6 ± 2.0 vs. 52.0 ± 2.6; *p* = 0.76, respectively). The StO_2_ level of the intestine on the oral side of the anastomosis one minute after the CRA ligation was significantly lower than immediately after the anastomosis (15.9 ± 6.0 vs. 52.0 ± 2.6; *p* = 0.006, respectively). There was no significant difference in the StO_2_ level between 1 min after and 30 min after the CRA ligation (15.9 ± 6.0 vs. 12.1 ± 5.3; *p* = 0.41, respectively).

**Conclusion:**

Novel oxygen saturation imaging endoscopy was safe and feasible to assess the anastomotic integrity in the experimental model.

## Background

Despite advances in surgical techniques and medical devices, anastomotic leakage and stricture remain one of the most dreaded complications in colorectal surgery. Anastomotic complications, such as postoperative morbidity and mortality, have a negative impact on short-term outcomes [1, 2]. In addition, they also affect long-term outcomes, such as survival in cancer patients [3] and quality of life [4].

Various risk factors have been reported to be associated with anastomotic leakage in colorectal surgery [1, 5]. Male sex, low anastomotic level (within 5 cm from the anal verge), and multiple firings with a linear stapler are the important factors for anastomotic leakage. Macroscopic intraluminal assessment of anastomotic completeness using an intraoperative colonoscopy and the air leakage test have been broadly used to check the incompleteness of the anastomosis during surgery [6–9]. It is also a vital procedure for avoiding anastomotic leakage to evaluate whether blood supply to the anastomotic site is sufficient [5, 10]. Recent technological advancement has successfully enabled real-time visualization of blood perfusion at the anastomotic site using indocyanine green fluorescence angiography (ICG-FA) in colorectal surgery [10]. ICG-FA has been widely used to evaluate anastomotic perfusion and it has been reported that it could reduce the incidence of anastomotic leakage in rectal surgery [10]. Conversely, there are also negative aspects such as the necessity of administering the fluorescent substance, possibility of ICG allergy, and the surgeons’ qualitative assessment.

If intraoperative endoscopy could enable the surgeon to assess blood perfusion of the anastomosis similar to that via a fluorescence scope, it would be a useful method for assessing not only blood perfusion, but also for obtaining a macroscopic view of the anastomotic line and the real-time status of the air leak test during colorectal surgery.

A novel endoscope system was therefore developed to assess the bowel perfusion in real time by capturing the tissue oxygen saturation (StO_2_) images [11, 12]. This system provides a repeated, quantitative assessment of bowel perfusion without the need for administering the fluorescent dye. Additionally, the system enables direct visualization of the anastomosis and allows the surgeon to conduct an air leak test to confirm mechanical completeness. The aim of the current study was to evaluate the feasibility of assessing the anastomotic integrity using a novel oxygen saturation imaging endoscopy in a porcine ischemia model.

## Methods

### Animals

The animals were obtained commercially from KAC Company Limited (Kyoto, Japan). A total of three pigs (domestic, female, crossbred with Large Yorkshire and Landrace, mean weight 47.2 ± 0.6 kg, 3 to 4 months of age) were used in the current non-survival study. The study protocol was approved by the Committee for Ethics of Animal Experimentation of the National Cancer Center (Japan) (K18-022). Experiments were performed according to the Guidelines for Animal Experiments (Science Council of Japan: Guidelines for Proper Conduct of Animal Experiments, 2006) and Animal Research: Reporting of In Vivo Experiments (ARRIVE) guidelines [13].

### Novel oxygen saturation imaging endoscope system

**Fig. 1:**
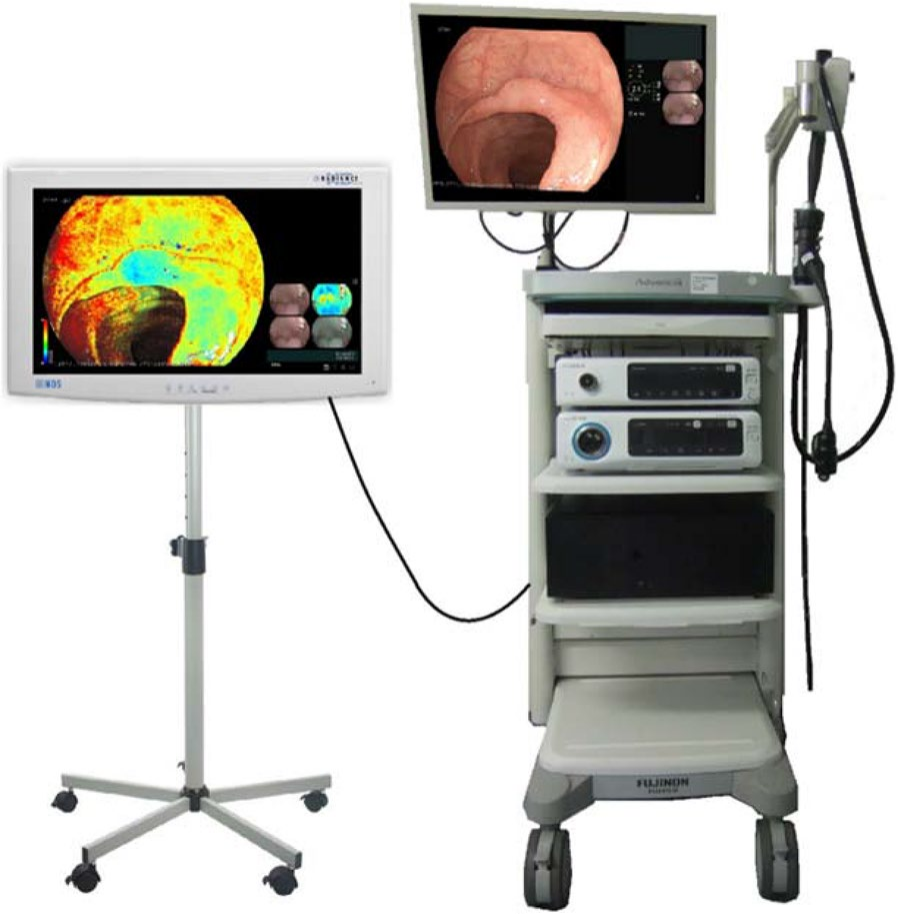
Novel oxygen saturation imaging endoscope system (printed from FUJIFILM Corporation, with permission)

**Fig. 2:**
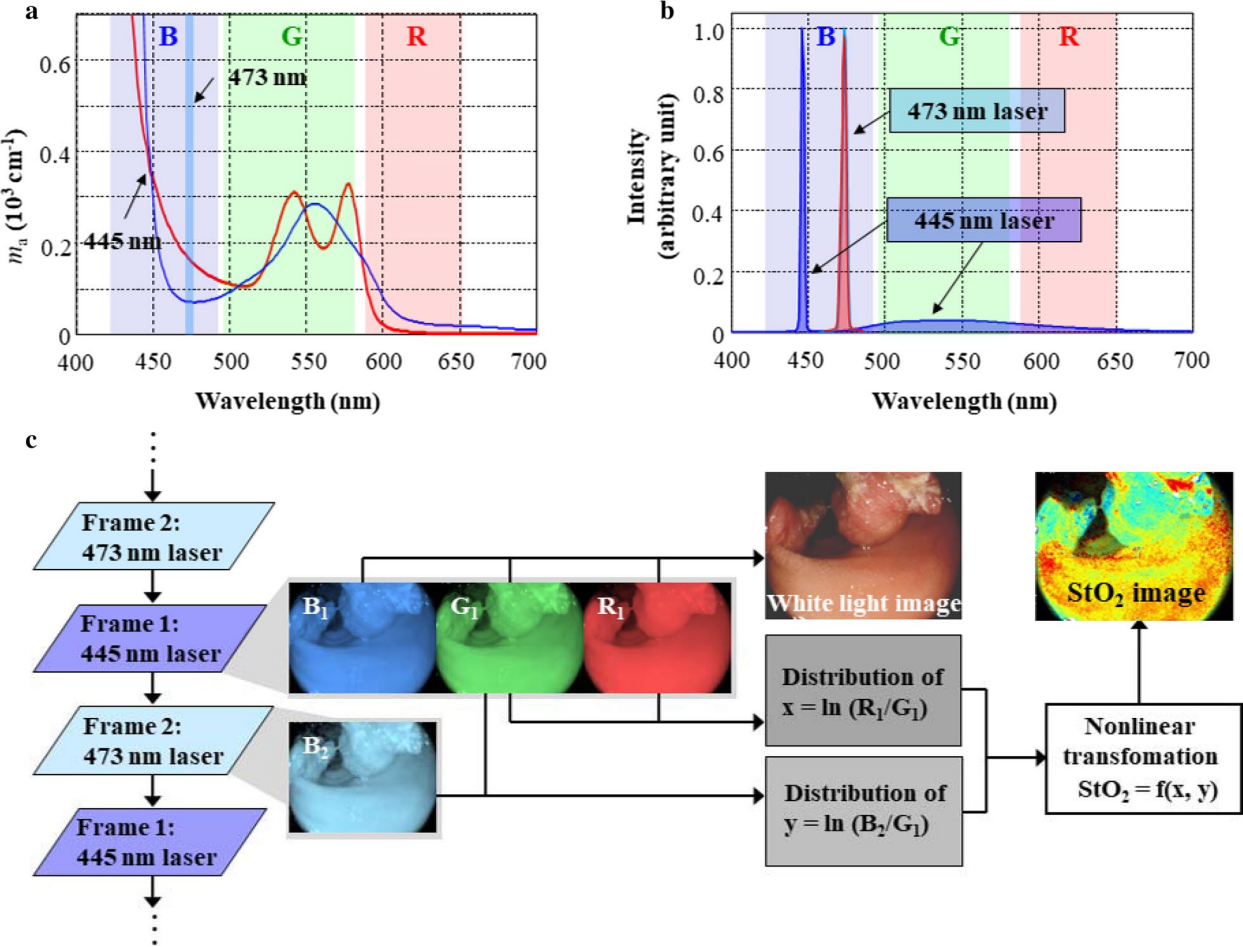
Mechanism of tissue oxygen saturation imaging

A novel endoscope system EP-0002 for gastroenterological flexible endoscopy (FUJIFILM Corporation, Kanagawa, Japan) was used to capture the StO_2_ images (Fig. 1) [11, 12]. The EP-0002 provides the conventional observation modes of white light imaging (WLI). Additionally, the system provides a mode of oxygen saturation imaging (OSI), where the images of StO_2_ distribution in the tissue being observed can be obtained in real time. The WLI and OSI modes can be displayed simultaneously on separate monitors (Fig. 1). The OSI technology can derive the StO_2_ images from the differences in the absorption coefficient in the visible light region between oxy- and deoxy-hemoglobin using a small number of wavelengths (Fig. 2a). The light source is equipped with three types of laser diodes (405, 445, and 473 nm) for illumination. The laser light is guided to the tip of the endoscope, which excites the phosphors mounted at the tip to create white fluorescent light. The 405 and 445 nm laser light is used in the WLI mode. In the OSI mode, the unit alternately emits 445 and 473 nm light synchronously with the video frames at a speed of 30 frames per second (fps). Images are acquired with a red–green–blue (RGB) color charge-coupled device mounted at the tip of the endoscope. The dominant wavelength ranges of the sensitivity of the RGB image sensor are overlaid on both graphs (Fig. 2b). The processor unit controls the whole system and performs image processing for the conventional modes of WLI. In the OSI mode, the processor unit acquires two types of images sequentially and alternately that correspond to the illumination induced by the 445 and 473 nm lasers, respectively (Fig. 2c). The processor unit alternately extracts frames of 445 nm light from the input video signals and uses them to display the WLI images on the processor’s monitor at a speed of 15 fps (Fig. 2c). At the same time, the processor unit transfers the video signals to the image processing (IP) unit, and the IP unit creates an StO_2_ image by using two consecutive frames corresponding to 445 and 473 nm illuminations (Fig. 2c). The StO_2_ images are displayed by assigning the StO_2_ level to the RGB display color of the monitor in 1% increments, with the StO_2_ level of 100% as red, 50% as yellowish green, and 0% as dark blue. The StO_2_ images are displayed on the IP’s monitor at a speed of 15 fps almost in real time (Fig. 1).

### Study protocol

After premedication with intramuscular injections of ketamine (10 mg/kg) and xylazine (2 mg/kg), general anesthesia was induced using 5% isoflurane. The airway was secured via endotracheal intubation, and general anesthesia was maintained with 1 to 3% isoflurane. The pigs were mechanically ventilated with the following settings: tidal volume, 10 to 15 ml/kg; respiratory rate, 12 breaths per minute; inspiratory to expiratory ratio, 1:2; positive end-expiratory pressure, 5 cmH_2_O; and fraction of inspired oxygen, 40%. Ringer’s lactate solution was administered intravenously through the auricular vein at 5 to 10 ml/kg/h. Heart rate, blood pressure, body temperature, and saturation of percutaneous oxygen (SpO_2_) were monitored throughout the procedure. An electrically heated mat was placed below each pig to prevent hypothermia.

**Fig. 3:**
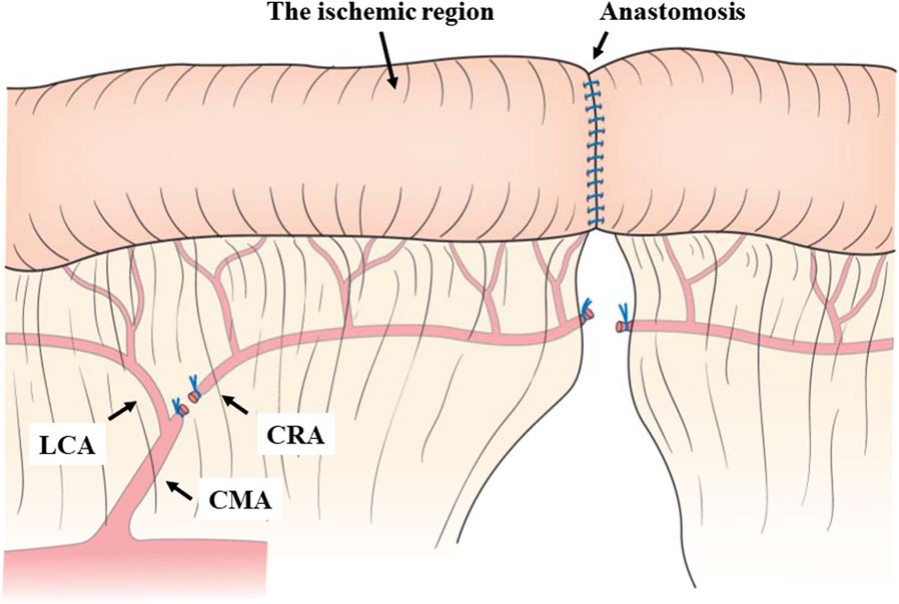
A schematic diagram of the porcine ischemia model. *CMA* caudal mesenteric artery, *CRA* cranial rectal artery, *LCA* left colic artery

Under general anesthesia, a midline laparotomy was performed. The marginal vessels of the rectum were dissected at the level of the planned anastomotic site, and the rectum was transected using a linear stapler (ECHELON FLEX ENDOPATH Stapler with a gold cartridge; Ethicon Endo-Surgery Inc, Cincinnati, OH, USA). The anvil was placed in the proximal side of the rectum, and end-to-end anastomosis was performed with the double stapling technique using a 25 mm diameter circular stapler (PROXIMATE ILS Curved Intraluminal Stapler; Ethicon Endo-Surgery Inc, Cincinnati, OH, USA). After the anastomosis, an air leak test was performed to check the completeness of the anastomosis via the endoscope. The root of the cranial rectal artery (CRA: equivalent to the superior rectal artery in humans) was dissected, and the porcine ischemia model was created in three pigs (Fig. 3). Prior to the assessment, the intestinal lumen was washed with saline to remove the stool. The contralateral side of the mesentery of the proximal rectum was marked with suture in each of the pigs. Bowel perfusion at the proximal side of the rectum was assessed intraluminally using a novel OSI endoscopy before and immediately after the anastomosis, as well as 1 min and 30 min after CRA ligation.

At the end of the procedure, the pigs were sacrificed by a rapid intravenous injection of a lethal dose of potassium chloride (75 to 150 mg/kg) while under deep general anesthesia using 5% isoflurane, and asystole was confirmed by an electrocardiogram.

### Quantitative evaluation of oxygen saturation imaging

Endoscopic procedures were recorded on videos and the stored still images were used to quantitatively evaluate the OSI with custom-made analysis software developed by FUJIFILM Corporation. A square region of interest was placed at three sites on the contralateral side of the mesentery of the proximal rectum in each of the pigs and each average value was calculated as the StO_2_ level. The StO_2_ levels were calculated at the following four time points: before and immediately after the anastomosis, and 1 min and 30 min after CRA ligation. The average StO_2_ levels of all three pigs were compared among the different time points.

### Statistical analysis

Statistical analyses were performed using JMP version 15.1.0 (SAS Institute, Cary, NC, USA). Values are presented as the mean ± standard deviation. For comparing the StO_2_ levels, a paired *t*-test was utilized. *p-*values < 0.05 were considered statistically significant.

## Results

Heart rate was 100 ± 20 beats per minute, mean arterial pressure was 58 ± 14 mmHg, body temperature was 36.6 ± 0.6 °C, and SpO_2_ was 97 ± 2.4, which were stable throughout the experiments. All procedures including endoscopic and surgical steps were uneventful. Establishment of the bowel ischemia model was systematically reproduced in all pigs.

**Fig. 4:**
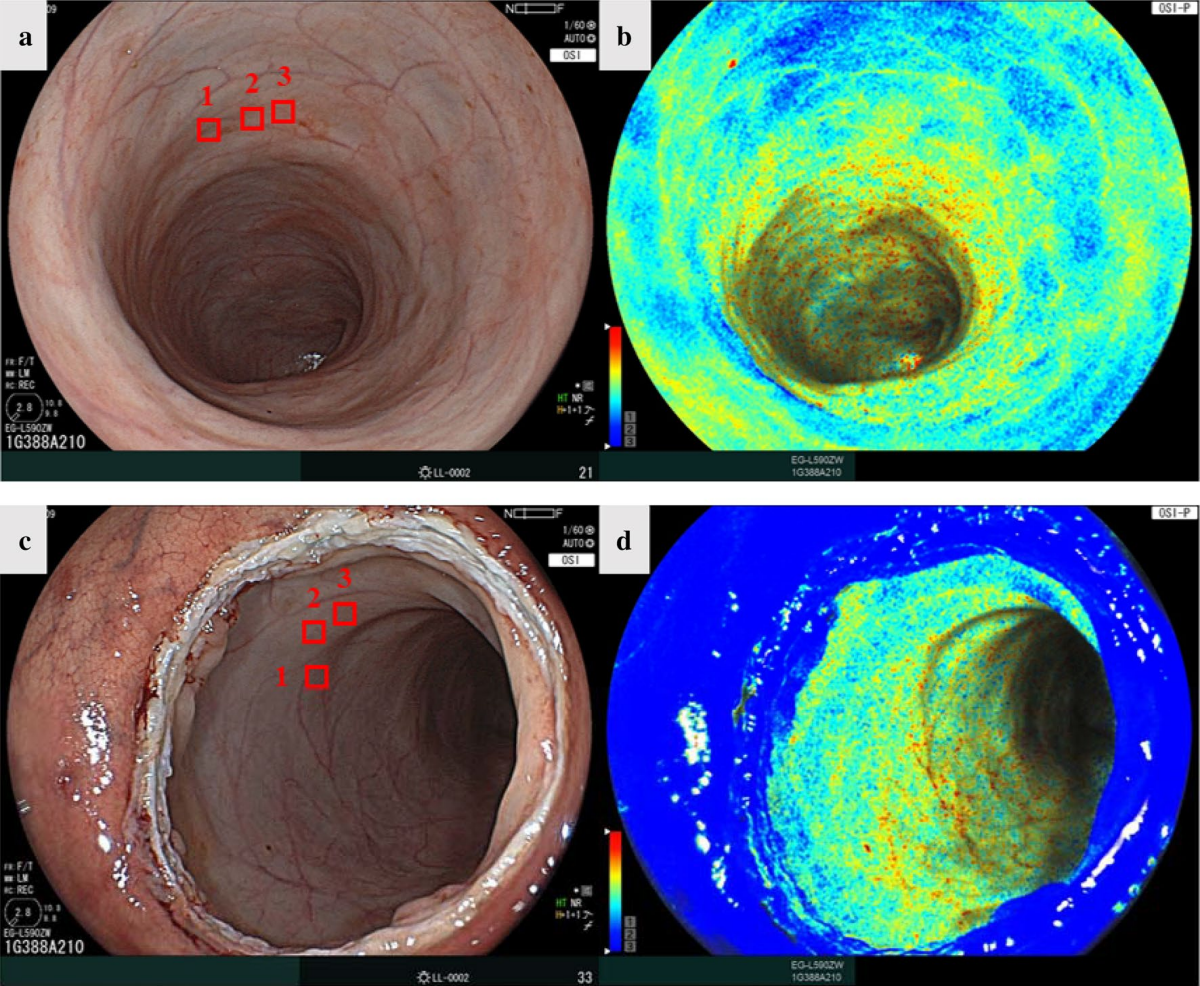
**a** White light image before the anastomosis. **b** StO_2_ map before the anastomosis. **c** White light image after the anastomosis. **d** StO_2_ map after the anastomosis. *StO*_*2*_ tissue oxygen saturation

**Fig. 5:**
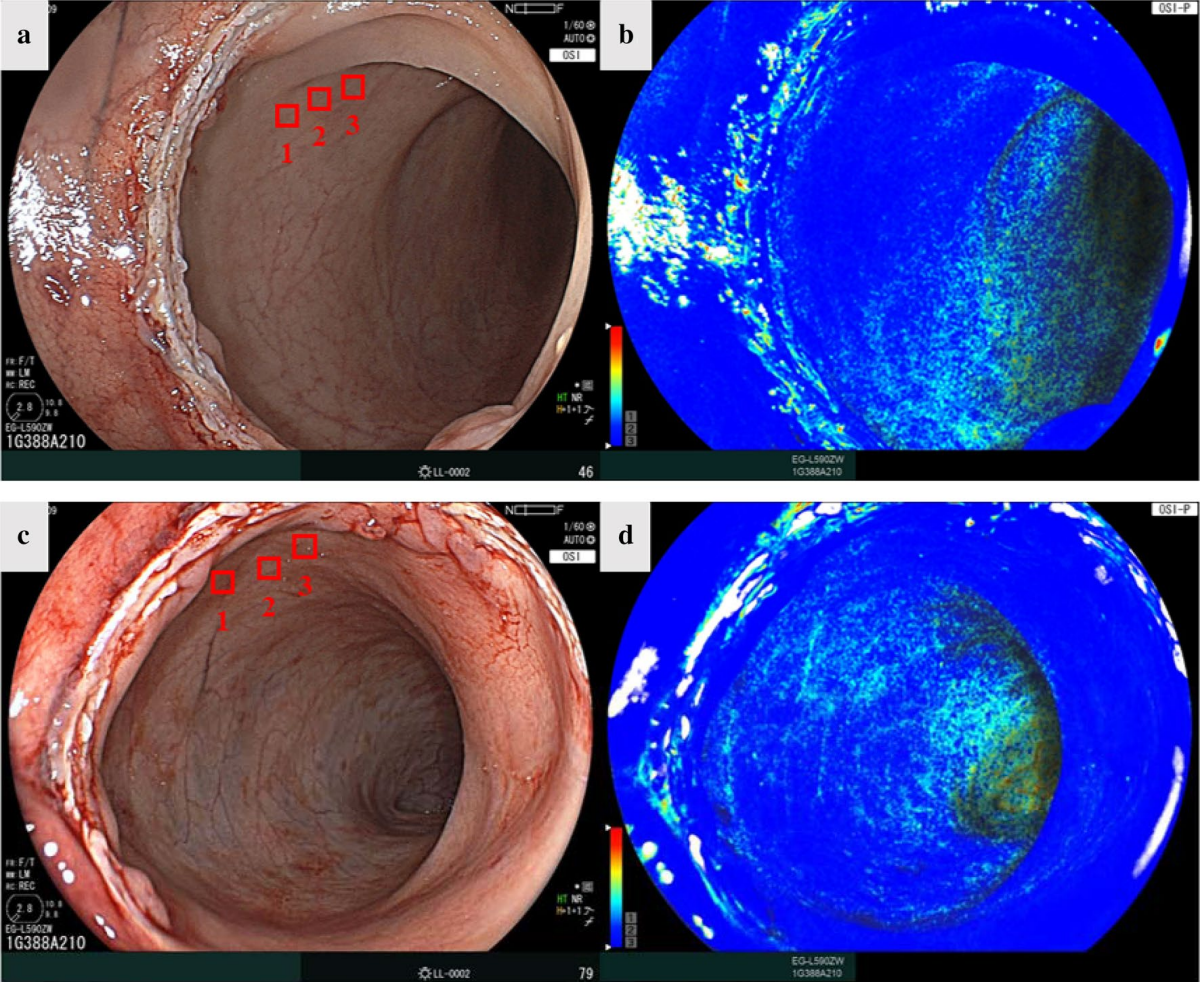
**a** White light image one minute after CRA ligation. **b** StO_2_ map 1 min after CRA ligation. **c** White light image 30 min after CRA ligation. **d** StO_2_ map 30 min after CRA ligation. *CRA* cranial rectal artery, *StO*_*2*_ tissue oxygen saturation

The status of the anastomosis was assessed intraluminally using the current endoscope after creating the anastomosis; there were no macroscopic findings of air leakage in any of the pigs. Oxygen saturation imaging was successfully performed in all animals. Before CRA ligation, the StO_2_ map of the proximal rectum showed a high StO_2_ value (Fig. 4). Immediately after CRA ligation, the StO_2_ map showed the appearance of a hypoxic area (Fig. 5). The hypoxic area was completely visible on the StO_2_ map and it corresponded to the ischemic regions. Bowel perfusion at the distal side of the rectum after dissection of marginal vessels showed a low StO_2_ value in all pigs.

Before the anastomosis, the mean StO_2_ level of the proximal rectum was 52.6 ± 2.0. Immediately after the anastomosis, the mean StO_2_ level was 52.0 ± 2.6. One minute after CRA ligation, the mean StO_2_ level decreased considerably to 15.9 ± 6.0; 30 min after CRA ligation, the mean StO_2_ level was 12.1 ± 5.3.

**Fig. 6:**
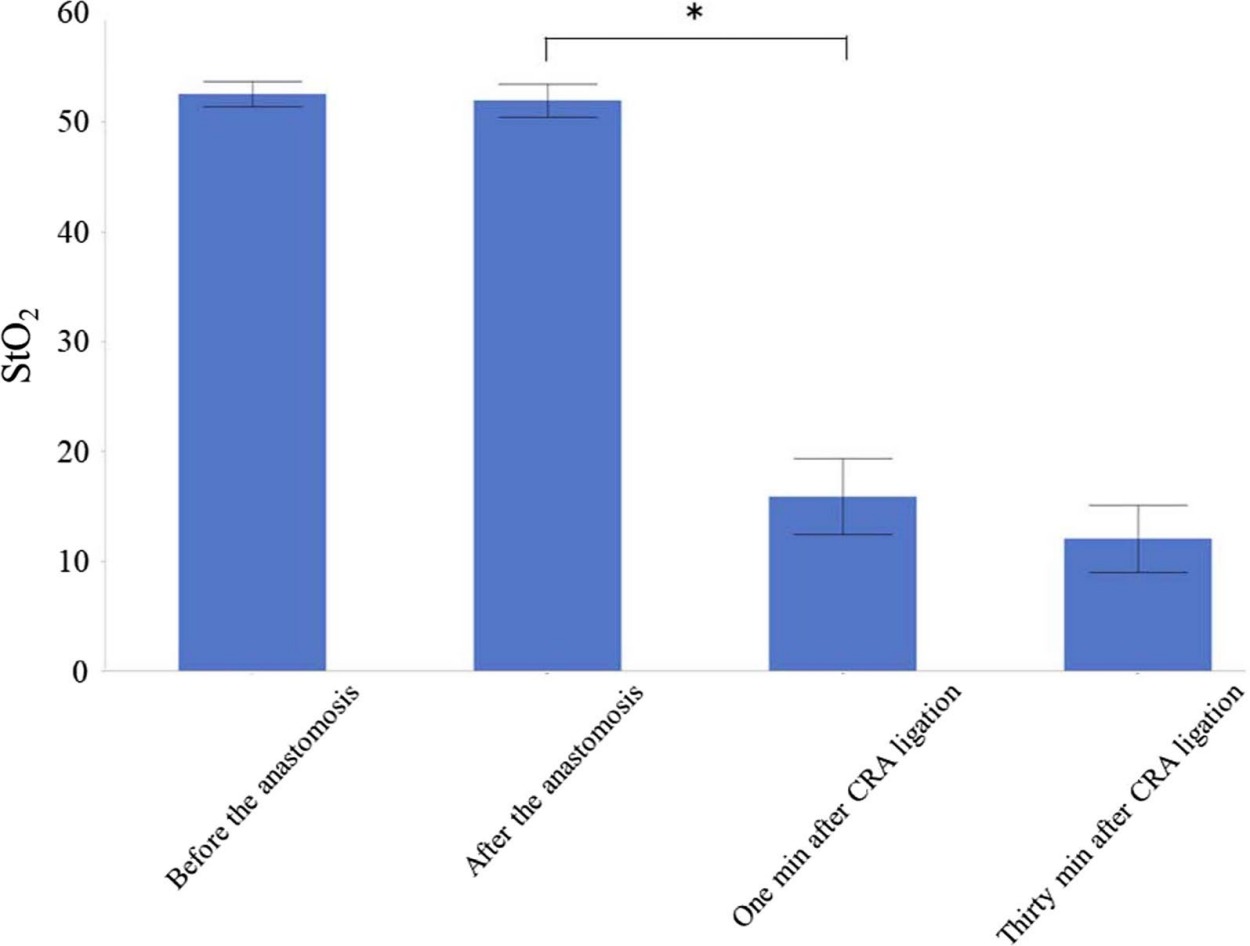
The average StO_2_ levels compared among the different time points. *Indicates a *p* value < 0.05. *CRA* cranial rectal artery, *StO*_*2*_ tissue oxygen saturation

There was no significant difference in the StO_2_ level before and immediately after the anastomosis (*p* = 0.76; Fig. 6). The StO_2_ level 1 min after CRA ligation was significantly lower than immediately after the anastomosis (*p* = 0.006; Fig. 6). There was no significant difference in the StO_2_ level between 1 and 30 min after CRA ligation (*p* = 0.41; Fig. 6).

## Discussion

Anastomotic leakage and stricture remain one of the most dreaded complications in colorectal surgery. To the best of our knowledge, this is the first study to evaluate the safety and feasibility of assessing anastomotic integrity using novel OSI endoscopy in an experimental porcine model. All procedures were safely performed without any adverse events, and the anastomotic assessment was successful in all animals. Regarding the vital signs, the standard deviation of heart rate and mean arterial pressure was relatively large, but did not affect the results. Using this system, the macroscopic statuses regarding the completeness and hemostasis achieved through the anastomosis could be directly checked not only by the intraluminal visualization, but also by an air leak test. Moreover, the bowel blood perfusion was repeatedly and quantitatively assessed without requiring the administration of fluorescent agents.

Anastomotic complications include patient-related, disease-related, and operative-related risk factors [1, 5]. Among these factors, operative-related risk factors can be controlled by the surgeons; anastomotic integrity is considered to be the most important factor to reduce anastomotic complications [6]. In particular, sufficient blood supply to the anastomosis and complete mechanical attachment (e.g., with staples) are essential in order to avoid the abovementioned complications [5, 6]. However, to the best of our knowledge, there is no medical device that can evaluate these elements simultaneously and in real time.

Recent image navigation technology has improved some postoperative outcomes; it is expected to expand to more clinical fields in the future. ICG-FA is an excellent example of this; it could be a safe and useful method to assess bowel perfusion intraoperatively in real time and reduce the incidence of anastomotic leakage by selecting an appropriate intestine with sufficient blood perfusion during colorectal surgery [10]. However, this method has several disadvantages. First, ICG-FA cannot confirm the macroscopic completeness of the anastomosis from inside the intestine. Secondly, ICG-FA requires the administration of ICG as a fluorescent substance; ICG takes a long time to be fully excreted from the body, which makes it difficult to repeatedly assess bowel perfusion. Third, the issue of ICG allergy persists. Fourth, the assessment of ICG-FA is qualitative and lacks objectivity. Intraoperative endoscopy would be a useful tool to assess the macroscopic status of the anastomosis and air leakage status along with enabling simultaneous intraluminal visualization [6–9]. In addition, it also enables therapeutic intervention to control the intraluminal bleeding [6]. However, to the best of our knowledge, there is no available endoscope system that can assess these factors simultaneously.

The novel endoscope system used in this study has been developed to assess bowel perfusion by deriving StO_2_ maps, which is one of the measures of tissue perfusion [11, 12]. Since this system does not require administration of the fluorescent substance, it can be performed anytime to assess bowel perfusion and can be performed repeatedly as well. In addition, a qualitative evaluation can be performed at a speed of 15 fps almost in real time. However, StO_2_ level cannot be acquired in real time because it is necessary to extract the stored still image and analyze it using dedicated analysis software in order to perform quantitative evaluation. Therefore, some improvements in the system are required for practical application.

This new endoscope system can also be used for the postoperative assessment of the anastomosis. Postoperative endoscopic evaluation of the anastomosis could enable the rapid use of the appropriate treatment [14].

## Conclusions

This novel OSI technology seems to be a promising method to assess not only the macroscopic status of, but also ischemic changes in the anastomosis immediately after ligation of the feeding vessel in an experimental model.

## Data Availability

The datasets used and/or analysed during the current study are available from the corresponding author on reasonable request.

## Abbreviations

CRA: Cranial rectal artery
Fps: Frames per second
ICG-FA: Indocyanine green fluorescence angiography
IP: Image processing
OSI: Oxygen saturation imaging
RGB: Red–green–blue
SpO_2_: Saturation of percutaneous oxygen
StO_2_: Tissue oxygen saturation
WLI: White light imaging
